# Validation of the Brazilian version of the Hinting Task and Facial Emotion Recognition Test (FERT-100) in patients with schizophrenia

**DOI:** 10.1590/1980-5764-DN-2021-0108

**Published:** 2022-05-23

**Authors:** Breno Fiuza Cruz, Amanda Margarida de Oliveira, Cristina Marta Del-Ben, Rhiannon Corcoran, João Vinícius Salgado

**Affiliations:** 1 Universidade Federal de Minas Gerais, Faculdade de Medicina, Departamento de Saúde Mental, Belo Horizonte MG, Brazil.; 2 Universidade Federal de Minas Gerais, Instituto de Ciências Biológicas, Programa de Pós-graduação em Neurociências, Belo Horizonte MG, Brazil.; 3 Universidade de São Paulo, Faculdade de Medicina de Ribeirão Preto, Departamento de Neurociências e Ciências do Comportamento, Ribeirão Preto SP, Brazil.; 4 University of Liverpool, Department of Primary Care and Mental Health, Liverpool, United Kingdom.; 5 Universidade Federal de Minas Gerais, Instituto de Ciências Biológicas, Departamento de Morfologia, Belo Horizonte MG, Brazil.

**Keywords:** Schizophrenia, Social Cognition, Validation Study, Theory of Mind, Esquizofrenia, Cognição Social, Estudo de Validação, Teoria da Mente

## Abstract

Social cognition is an especially relevant domain in schizophrenia due to its association with functional impairment. However, we still do not have studies that have validated instruments with internationally established psychometric qualities for the Brazilian population.

**Objectives:**

This study aimed to present psychometric qualities and contribute to the validation of the Brazilian version of the Hinting Task and Facial Emotion Recognition Test (FERT-100).

**Methods:**

A total of 104 stabilized patients living in the community diagnosed with schizophrenia and 89 controls were evaluated. We assess the psychometric properties of Hinting Task and FERT-100 for discriminant construct validity, divergent construct validity, convergent construct validity, concurrent criterion validity, and reliability.

**Results:**

There is a statistically significant difference between patients and controls regarding social cognition (Hinting Task: Z=6.85, p<0.001; FERT-100: t=4.88, p<0.001). The main predictors of variation in social cognition were the neurocognitive domains. The associations between social cognition tests and other studied variables are similar to what is found in the literature. Social cognition maintains correlation with functional capacity even when neurocognition is taken into account.

**Conclusions:**

The validity of the Brazilian version of Hinting Task and FERT-100 can be determined, since the relationship of these tests with other clinical variables is similar to that observed in the literature.

## RESUMO

A cognição social é um domínio especialmente relevante na esquizofrenia devido à sua associação com o comprometimento funcional. No entanto, ainda não temos estudos que validaram instrumentos com qualidades psicométricas internacionalmente estabelecidas para a população brasileira.

**Objetivos:**

Apresentar as qualidades psicométricas e contribuir para a validação da versão brasileira do *Hinting Task* e do Teste de Reconhecimento de Emoções Faciais (FERT-100).

**Métodos:**

Foram avaliados 104 pacientes estabilizados residentes na comunidade com diagnóstico de esquizofrenia e 89 controles. Avaliou-se as propriedades psicométricas do *Hinting Task* e FERT-100 para validade de construto discriminante, validade de construto divergente, validade de construto convergente, validade de critério concorrente e confiabilidade.

**Resultados:**

Houve uma diferença estatisticamente significativa entre pacientes e controles quanto à cognição social (*Hinting Task*: Z=6,85; p<0,001. FERT-100: t=4,88; p<0,001). Os principais preditores da variação na cognição social foram os domínios neurocognitivos. As associações entre os testes de cognição social e outras variáveis estudadas são semelhantes às encontradas na literatura. A cognição social mantém correlação com a capacidade funcional mesmo quando a neurocognição é levada em consideração.

**Conclusões:**

A validade da versão brasileira do *Hinting Task* e do FERT-100 pode ser determinada, pois a relação desses testes com outras variáveis clínicas é semelhante à observada na literatura.

## INTRODUCTION

Social cognition is defined as the mental operations behind social interactions, which include the human capacity to perceive intentions and dispositions of others. In short, this means how people think and form impressions of people^1–3^. Although the processing of socially relevant information also depends on neurocognition (e.g., attention or memory), it has been shown that neurocognition and social cognition are dissociable domains^3^.

The domains most studied in this broad construct are theory of mind (ToM) and emotion processing (EP)^4–6^. Emotion processing refers to perception and use of emotions and usually involves tests that evaluate recognition of emotional expressions on faces^7^. Human face is one of the richest sources to accurately infer other people’s mental and emotional state. This information is relevant to the observer on how to behave in the social environment^8^. ToM is conceptualized as a system of references that enables comparisons between the internal, subjective world, and the external world, of others^9,10^. According to the study by Premack and Woodruff^11^, an individual has a ToM if he imputes mental states to himself and to others. Additionally, a system of inferences of this nature is properly seen as a “theory” because such states are not directly observables and the system can be used to make predictions (theorizations) about the behavior of others. The ToM tests generally rely on short verbal reports and/or interactions between characters who have a false belief or use irony or indirect speech.

Several studies and meta-analysis^12^ suggest that deficits in EP and ToM are present in patients with schizophrenia and their first-degree relatives. These deficits are stable over time and do not respond to antipsychotic treatment. Thus, social cognition can be considered an endophenotype of schizophrenia^7,13^.

A meta-analysis by Savla et al.^1^ demonstrated that patients with schizophrenia have impaired social cognition compared to controls, with an effect size (g) of 0.96 for ToM and 0.89 for EP. And although deficits in social cognition are moderately correlated with neurocognition, negative, and disorganized symptoms, these deficits remain relevant even when considering such factors, with social cognition impaired in schizophrenia even in stabilized patients^1^.

Importantly, social cognition is strongly and independently related to functional performance^2,4–6^.

The Social Cognition Psychometric Evaluation (SCOPE) initiative^14^ evaluated several social cognition tests available based on (1) test-retest reliability, (2) utility as a repeated measure, (3) relationship with functional performance, and (4) practicality and tolerability. This initiative found that only one ToM test, one Hinting Task, and two facial emotion recognition tasks had the best psychometric properties and were recommended for use in clinical trials. Unfortunately, we do not yet have instruments that assess ToM and EP in patients with schizophrenia validated for the Brazilian population.

The objective of this study was to adapt to Brazilian Portuguese and analyze some of the psychometric properties of the Hinting Task and the Facial Emotion Recognition Task (FERT-100) in patients with schizophrenia.

## METHODS

### Participants

A total of 104 stabilized schizophrenia outpatients, aged between 18 and 65 years, participated in this study. Patients diagnosed with schizophrenia undergoing outpatient treatment at the Raul Soares Institute (Belo Horizonte – MG) and at the psychiatry outpatient clinic of the city of Nova Lima (MG) were invited to participate in the study. A structured interview using the MINI-plus was used to confirm the diagnosis^15,16^. Patients with alcohol or any drug dependence (except nicotine), history of neurological disease, mental retardation, or brain trauma were excluded. Stabilization was defined by scoring 19 or less in the Positive and Negative Syndrome Scale (PANSS) positive subscale (see below) and 4 or less in any item of this subscale^17^.

Schizophrenia patients were matched for gender and age to 89 controls. Students from the youth and adult education program from two municipal schools in Belo Horizonte (MG) were selected through written and/or oral invitation to participate as controls. Criteria for inclusion and exclusion of controls were as follows: age over 18 years and under 65 years, having no history of neurological disease, mental retardation or brain trauma, and not having any pathology of axis one of the *Diagnostic and Statistics Manual of the American Psychiatric Association* (*DSM-IV*), confirmed by MINI-plus^15,16^.

All invited participants were instructed on the study design and its objectives. Those who agreed to participate signed an informed consent form, according to the local ethics committee.

### Evaluation scales

#### Clinics/psychopathology

The PANSS^18,19^ and the Calgary Depression Scale for Schizophrenia (CDSS)^20,21^ were used to assess positive/negative symptoms and depressive symptoms, respectively. The Positive and Negative Syndrome Scale is composed of seven items in each subscale (positive and negative symptoms). A score of “1” is given in the absence of symptoms, and a score of “7” is given to the most severe symptomatology. Thus, both subscales have a minimum score of 7 and a maximum score of 49^18,19^. The CDSS is composed of nine items. The score is given so that “zero” corresponds to the absence of the evaluated symptom and “3” to its presence in maximum severity. The score 21 is the maximum score possible.

#### Neurocognition

The Brief Assessment of Cognition in Schizophrenia (BACS) was used to assess neurocognition^17,22,23^. This instrument is an easy and fast to administer neuropsychological battery developed to assess the main cognitive domains impaired in schizophrenia: verbal memory (measure: number of words remembered in any order-score: 0–75), working memory (digit span test — measure: number of correct answers; score: 0–28), motor speed (token motor task — measure: number of tokens correctly placed in the container during 1 min; score: 0–100), verbal fluency (semantic and phonetic — measures: number of words generated), processing speed (symbol coding — measure: number of correct answers; score: 0–110), and reasoning/problem solving (Tower of London — measure: number of correct answers; score: 0–22)^17^. To compare the mean scores presented by participants with schizophrenia in relation to controls, we calculated the Z-score. Its calculation consists of subtracting the mean score obtained from participants with schizophrenia in relation to controls and dividing the result by the standard deviation of controls.

#### Social cognition

##### Hinting Task (Brazilian version)

This task was conceived to assess subjects’ ability to infer implicit intentions. It comprises 10 small stories, each one with a very obvious hint about what one of the character implicitly meant. If the participant gives a correct answer about the character intention, it scores two points. Otherwise, an even more obvious hint is given. In this phase, a correct and a wrong answer score one and zero point, respectively. The final score ranges from zero to 20. All stories are read aloud with the appropriate prosody^10^. The instrument was translated to Brazilian Portuguese and back-translated to English with the supervision of the original author (R. Corcoran). A pilot study with 20 people with 9 years of schooling was carried out in order to assess the understanding of the stories and instructions. After minor modifications, the final version was applied to the study participants (see Supplementary Material I for task full final version).

##### Facial Emotion Recognition Task

In this task, participants are asked to recognize emotions in 100 black and white pictures of Caucasians’ faces from Ekman catalogue of facial emotion^24^. Each picture was presented in a 15-inch computer screen for 0.5 s. Participants had 2 s to guess, by pressing a computer key, which emotion best describe the one they saw in the picture. The emotions are fear, anger, disgust, sadness, surprise, and happiness. A total of 96 pictures of these emotions were randomly distributed in the same amount, in four different intensities (30, 50, 70, and 100% of intensity), to be present to each patient. There were also four pictures with faces without any emotion, to which patients should guess NEUTRAL. The task was run in a Matlab program, version R2007a.

#### Functional capacity assessment

The UCSD Performance-based Skills Assessment (UPSA) assesses the ability to perform tasks typical of everyday life in community. It comprises five domains: comprehension and planning (score range: 0–27), finance (score range: 0–10), communication (score range: 0–9), mobility (score range: 0–6), and home care (score range: 0–4). Each domain is scored as follows: the number of points obtained is divided by the total possible points and this result is multiplied by 20. Score range is 0–100. The Brazilian-Portuguese version has shown good psychometric properties to assess functional capacity^25,26^.

### Validation of social cognition tests

We assess the psychometric properties of Hinting Task and FERT-100 as follows:

- For discriminant construct validity, we compared the results obtained between patients and controls.
- For divergent construct validity, we looked at associations of social cognition tests with each other, sociodemographic data, symptomatology, and neurocognition.
- For convergent construct validity, we looked at associations between tests of social cognition and functional capacity.
- For reliability, we use internal consistency (Cronbach’s alpha).
- For concurrent criterion validity, we compared our results with the original test (Hinting task) and with the literature (Hinting Task and FERT-100), in “DISCUSSION” section.

### Design

Each participant was tested in one session of about one and a half hour. The instruments were applied as follows: sociodemographic questionnaire, MINI-plus, PANSS, BACS, Hinting Task, FERT-100, and UPSA.

### Statistical analysis

The SPSS software (IBM), version 20, was used for the statistical analysis of the data. Parametric distribution of all variables was verified using the Kolmogorov-Smirnov test. Pearson’s (for parametric data) and Spearman’s (for nonparametric data) correlations were made between the variables of interest. For comparisons between patients and controls, Student’s *t*-test or Mann-Whitney U test was used, depending on the normality of data. For comparison between gender of patients and controls, χ^2^ test was used. Hinting Task and FERT-100 internal consistency was calculated using Cronbach’s alpha. ANOVA test was also used to compare the number of correct answers to different intensity of emotions, assessed by the FERT-100. Multiple linear regression analysis was performed to assess predictors of social cognition tests. The score obtained in Hinting Task was normalized using reflected logarithm. This transformation allows normalization of data with a negative asymmetric distribution, using the following formula: Transformed data = log10 (highest value obtained in the test + 1 − original data)^27^. It is a trend in literature that just carrying out tests of significance of the null hypothesis is not enough to compare difference in means of two or more variables. Estimation techniques such as effect size and confidence intervals are increasingly being used to observe the magnitude of difference between two variables and thus establish the real importance of an intervention^28^. Thus, Hedge’s g effect size was calculated.

## RESULTS

### Sample

Sociodemographic and clinical data are shown in Table 1. There was no statistically significant difference between mean age, gender, and education between patients and controls. Patients have low scores on the subscale of positive symptoms of PANSS and depressive symptoms on Calgary and low-to-moderate scores on the subscale of negative symptoms.

**Table 1:** Sociodemographic and clinical data for patients and controls.

		Patients (n=104)	Controls (n=89)	Statistical test	p-value
Mean age (SD)		41.99 (12.07)	40.23 (11.58)	T=-1.07	0.286
Gender, male (%)		59 (56.6)	49 (55.1)	X^2^=0.53	0.818
Education years (SD)		7.12 (4.19)	7.77 (2.46)	Z=1.72	0.085
Antipsychotic dose à chlorpromazine equivalent/mg (SD)		316.05 (216.93)			
PANSS (SD)	Positive	9.94 (2.86)			
	Negative	18.57 (6.86)			
	General	26.05 (6.46)			
	Total	54.35 (12.78)			
Calgary		1.98 (2.27)			

T: Student’s *t*-test; Z: Mann-Whitney U test; SD: standard deviation; PANSS: Positive and Negative Syndrome Scale.

### Discriminant construct validity

#### Comparison between patients and controls

##### Hinting Task

As distribution of the Hinting Task result does not follow a normal distribution, the Mann-Whitney U test was performed to compare the score of patients and controls. As can be seen in Table 2, there is a statistically significant difference between patients and controls on this task.

**Table 2:** Difference between controls and patients: social cognition.

Social cognition tasks	Patients (n=104) Mean (SD)/median	Controls (n=89) Mean (SD)/median	Statistical test	p-value	Cronbach’s alpha
Hinting Task	13.89 (3.41)/ 15	17.11 (1.98)/17	Z=-6.85	<0.001	0.68
FERT-100	34.59 (13.00)	44.17 (10.92)	T=4.88	<0.001	0.87

SD: standard deviation; FERT-100: Hinting Task and Facial Emotion Recognition Test.

Calculating effect size (Hedge’s g) for difference between the mean of correct answers in tests, it is observed that Hinting Task obtained a value of g = 1.2. This means that there is an overlap between the scores of patients and controls of 37%. With normalization of the results obtained in Hinting Task by calculating the reflected logarithm of the scores obtained in this test^27,29^, the effect size remains practically unchanged (g=1.16). Thus, despite caution when analyzing the effect size for Hinting Task, data normalization revealed very similar values.

##### FERT-100

In FERT-100, Student’s *t*-test was used to compare the score of patients and controls as these data obey a normal distribution. In this case, there was a statistically significant difference between mean total correct answers between patients and controls (Table 2).

FERT-100 presented a Hedge’s g value=0.8. This means that there is an overlap between the scores of patients and controls of 53%.

#### Analysis of scale items

##### Hinting Task

A comparison between the 10 stories of Hinting Task was also performed using the Mann-Whitney U test (Supplementary Material II Table 1). It is observed that the scores of patients and controls differ in all histories, with the exception of story 02, whose p-value is 0.065 (Table 3). Removing story 02, Cronbach’s alpha goes to 0.66, so it was decided to keep the original 10 test stories in other analyzes of this work.

**Table 3:** Social cognition correlations.

	Age	Years (education)	PANSS		Calgary	FERT-100	UPSA
			Positive	Negative			
Hinting Task	-0.08	0.211	-0.67	-0.241*	-0.248*	0.288**	0.518**
FERT-100	-0.13	0.38**	0.09	-0.134	0.15		0.548**

PANSS: Positive and Negative Syndrome Scale; FERT-100: Hinting Task and Facial Emotion Recognition Test;

* p<0.05;

** p<0.01.

##### Hinting Task and Facial Emotion Recognition Test

An assessment of concordant correct answers between patients and controls, in each of the types and intensities of emotions, was performed in FERT-100 (Supplementary Material II Table 2). Happiness was the emotion with the highest mean of concordant correct answers between patients and controls, and fear was the least. A higher level of intensity of emotions was accompanied by greater accuracy, both in patients and controls, as observed when performing an ANOVA of repeated measures (patients: F=230.142; controls: F=259.307; p<0.001).

A comparison was also made between the mean scores of patients and controls regarding the type and intensity of emotions observed during the performance of the FERT-100, using Student’s t-test (Supplementary Material II Table 2). The mean of correct answers differs between patients and controls among all levels of intensity of emotions. As for the type of emotions, all means of correct answers differed between patients and controls, except fear and sadness. When considering the 95% confidence interval between the averages, in addition to fear and sadness, the intensity of 30% of the emotions also shows an intersection between the confidence intervals of patients and controls (Supplementary Material II Table 2).

#### Divergent construct validity

##### Associations with sociodemographic data, symptomatology, and neurocognition

As can be seen in Table 3, social cognition tests do not correlate with age and antipsychotic dose in patients. FERT-100 test correlated with education years (r=0.380; p<0.01) in this sample.

There was no correlation between sociodemographic data and social cognition in the controls.

Hinting Task correlated with negative PANSS (rho=-0.241, p<0.05) and Calgary (rho=-0.248; p<0.05). FERT-100 was not related to symptoms.

The Hinting Task correlates weakly with FERT-100 (rho=0.288, p<0.01), which would be expected, as both assess social cognition, but different domains (ToM and perception of emotions, respectively). The Hinting Task and FERT-100 also correlate weakly or moderately across all domains of general cognition, with the exception of motor speed (Token motor task), as shown in Table 4. The mean BACS Z-score for patients was -1.08, replicating result of meta-analysis^30^.

**Table 4:** Social cognition and neurocognition correlations.

	Verbal memory	Digit span	Token	Verbal fluency	Symbol	T. London	Z-score
Hinting Task	0.397**	0.323**	0.125	0.386**	0.383**	0.314**	0.451**
FERT-100	0.366**	0.355**	0.135	0.411**	0.443**	0.540**	0.502**

FERT-100: Hinting Task and Facial Emotion Recognition Test;

** p<0.01.

Multiple linear regressions were also performed to analyze predictors of social cognition scores tests. All variables that showed statistically significant correlations with social cognition tests were evaluated. Regarding Hinting Task, only verbal fluency and working memory (digit span task) remained in the model, together explaining 26% of the variation (22% for verbal fluency and 4% for working memory) (Supplementary Material II Table 3). Using BACS Z-score instead of cognitive domains in isolation, the model had a lower prediction. Normalization of data through reflected logarithm did not bring significant changes to the model. Despite this, these data should be analyzed with caution, since Hinting Task score does not have a normal distribution.

Linear multiple regression for FERT-100 found that the BACS Z-score explains 37% of test variation (Supplementary Material II Table 4). In this case, the model with BACS Z-score brought a greater prediction to the FERT-100 than use of cognitive domains separately. Other variables that showed significant simple correlations with the test did not maintain significant statistical value (p<0.05) in multiple regression.

#### Convergent construct validity

Social cognition tests also correlate moderately/strongly with functional capacity, assessed by UPSA-BR (Hinting Task: rho=0.52; p<0.001; FERT-100: r=0.55; p<0.001). Social cognition tests and UPSA correlation remains significant even when result is controlled taking neurocognition into account (r=0.42; p=0.002 for Hinting Task and r=0.27; p=0.05 for FERT-100). And when social cognition tests’ scores are considered, the correlation between neurocognition and UPSA-BR loses strength, going from r=0.65 to 0.52 (p<0.001) when controlling the result considering Hinting Task and to 0.39 (p=0.003) when controlling the result considering FERT-100.

#### Reliability

As for internal consistency, Cronbach’s alpha was 0.68 for the Hinting Task, which approaches the appropriate value of 0.8 for use as a research tool^14^ and is also a value very similar to that found by Gil et al.^31^ (0.69), who validated the Hinting Task for Spanish and identical to the value found by Pinkham et al.^14^. The Cronbach’s alpha for FERT-100 was 0.87.

## DISCUSSION

The concurrent criterion validity of a test may be assessed by comparing the results obtained with those seen in literature^32^. The means and standard deviations of Hinting Task found in our study were very similar to the study by Pinkham et al.^33^ (patients: 13.89±3.41 vs. 13.59±3.87; controls: 17.11±1.98 vs. 16.82±2.05). Another similarity was between the correlation Pinkham et al.^33^ also found an association of Hinting Task with UPSA (r=0.462) very similar to the one found in the present study (r=0.518), both with p<0.001.

The effect sizes for difference between patients and controls regarding ToM and EP found in this study (1.2 and 0.8, respectively) are similar to that found in meta-analysis by Savla et al. (0.96 for ToM and 0.89 for EP)^1^. They are also very similar to the effect size observed by Pinkham et al.^33^, who observed an effect size for Hinting Task d=1.06. These same group also demonstrated that Hinting Task and emotion recognition tests show the best psychometric qualities among several evaluated social cognition tests and recommend them for use in clinical trials^14^. The emotion recognition tests evaluated by these authors were Penn Emotion Recognition Task (ER-40) and Bell Lysaker Emotion Recognition Task (BLERT). The ER-40 uses 40 static pictures and just 4 emotions. This instrument was only recommended for use after modifications that allowed it to increase its ability to predict functional performance. BLERT uses the same seven emotions as the FERT-100. Through 21 videos, a male actor provides information about his emotions through facial mimicry, tone of voice, and body movements. This instrument has been indicated for use in clinical trials without modifications. It is observed that the emotion recognition test of the present study (FERT-100) presents characteristics of both tests analyzed above, being more comprehensive than the ER-40 and simpler than the BLERT, eliminating the need for video. These characteristics proved to be valid, since the FERT-100 was able to correlate with measurement of functional capacity.

Another aspect that reflects the psychometric qualities of the Hinting Task is its discriminative validity with the emotion recognition test. The correlation between them is weak (r=0.29; p<0.01), which is expected, since they assess different subdomains of social cognition (ToM and EP, respectively)^34^. This finding is supported by Lysaker et al.^35^ and Hagiya et al.^36^, who found a correlation between the Hinting Task and a facial expression recognition test similar to that found in our study (r=0.33 and r=0.34, respectively).

Mehta et al.^37^ found that neurocognition predicts about 19% of the variation in ToM and 39% of the variation in emotion recognition in remitted patients with schizophrenia. These results are similar to this study, whose multiple regression demonstrated that neurocognition explains 26% of the variation in Hinting Task and 37% in FERT100 (Supplementary Material II Tables 3 and 4). The meta-analysis by Ventura et al.^38^ also confirms that correlations between social cognition and neurocognition are mostly moderate and consistent.

This study corroborates the study by Brown et al.^39^, in the findings that Hinting Task is associated with negative symptoms, but not with positive symptoms, and that there are no associations between symptomatology and facial emotion recognition tests. Brown’s study did not assess depressive symptoms, which correlate weakly with Hinting Task in this study. It is worth remembering, however, that both negative and depressive symptoms did not enter Hinting Task’s multiple linear regression model.

This study showed that fear and sadness were the emotions in which there were no significant differences between patients and controls in the FERT-100. It was also found that happiness is the emotion with the highest number of correct answers and fear the least, in both patients and controls. In addition, the increase in intensity in the expression of emotions increases the number of correct answers. These results are similar to those found by Hargreaves et al.^40^, who also demonstrated that happiness and fear are the emotions with the highest and lowest average scores, respectively, as well as that accuracy increases with the intensity of emotions. However, in this study, the emotion that did not differ in correct answers between patients and controls was surprise. The finding of this study that fear is an emotion with less identification in controls and patients is also supported in the literature^41,42^.

A very relevant finding of this work is that tests of social cognition correlate with measurement of functional capacity (UPSA), even when neurocognition is considered, which is also demonstrated in works of Pinkham et al.^14,33^. This finding reinforces the importance of social cognition tests, as this cognitive domain is an essential factor to understand, propose, and evaluate interventions aimed at the full functional recovery of patients with schizophrenia.

We are not aware of any study that comprehensively validated specific social cognition tests for patients with schizophrenia in the Brazilian population. The work by Fonseca et al.^43^ assessed the psychometric assessment of MATRICS consensus cognitive battery (MCCB) for the Brazilian population. This cognitive battery contains an instrument for assessing social cognition, the Mayer–Salovey–Caruso Emotional Intelligence Test (MSCEIT-ME): Managing Emotions. This is not a test that specifically and comprehensively assesses domains of ToM and EP. The work by Negrão et al.^44^ adapted and validated the “Faux Pas Recognition Test”^45^, considered a test that assesses ToM, initially used to assess this domain in patients with frontal lobe lesions. This test has not been evaluated in the work by Pinkham et al., and thus, we cannot infer its employability as a measure that relates to functional performance in schizophrenia, for example.

The main limitation of the study was the inability to compare the results obtained with the application of the Hinting Task and FERT-100 to social cognition scales already validated for schizophrenia in the Brazilian population.

In summary, this study confirms data from literature that patients have deficits in social cognition compared to controls, that social cognition is related to neurocognition and functional performance, providing an additional explanation for neuropsychological tests in relation to functional impairment. In addition, Hinting Task weakly correlates with negative symptoms and facial emotion recognition. Thus, this evidence suggests that the instruments used are valid tools to assess social cognition in schizophrenia.

Impairments in social cognition are fundamental characteristics of schizophrenia and are closely linked to impaired functional performance that occurs in this mental disorder^5^. There are few duly validated tests that assess social cognition for the Brazilian population that suffers from this disorder, limiting the assessment of this important construct in this population. In this study, social cognition tests (Hinting Task and FERT-100) showed psychometric qualities that give validity to their use in Brazilian population with schizophrenia.
